# Exoscopic Minimally Invasive Open-Door Laminoplasty for Cervical Myelopathy: A Technical Note and Preliminary Analysis of Clinical Outcomes during the Acute Postoperative Period

**DOI:** 10.3390/jcm13082173

**Published:** 2024-04-10

**Authors:** Kentaro Yamane, Wataru Narita, Shinichiro Takao, Kazuhiro Takeuchi

**Affiliations:** 1 Department of Orthopaedic Surgery, National Hospital Organization Okayama Medical Center, 1711-1, Tamasu, Kita-ku, Okayama 701-1192, Okayama, Japan; mechamecha_su_te_ki@yahoo.co.jp (S.T.); takeuchi.kazuhiro.qr@mail.hosp.go.jp (K.T.); 2 Department of Orthopaedic Surgery, Kameoka Municipal Hospital, 1-1, Shinonoda, Shino-cho, Kameoka 621-8585, Kyoto, Japan

**Keywords:** axial pain, cervical myelopathy, exoscope, exoscopic minimally invasive open-door laminoplasty, open-door laminoplasty, spinal treatment

## Abstract

**Background/Objectives:** Expansive open-door laminoplasty results in favorable clinical outcomes for cervical myelopathy. However, some postoperative complications associated with surgical invasiveness, such as axial neck pain and kyphosis, have not been resolved. The use of an exoscope, which is a recently introduced novel magnification tool, allows for traditional open-door laminoplasty with minimal invasiveness. Therefore, we propose the use of exoscopic minimally invasive open-door laminoplasty (exLAP) and present its clinical outcomes during the acute postoperative period. **Methods**: A total of 28 patients who underwent open-door laminoplasty at C3–C6 were reviewed. Of these patients, 17 underwent exLAP (group M) and 11 underwent conventional Hirabayashi open-door laminoplasty (group H). Outcomes were evaluated using numerical rating scale (NRS) scores for neck pain and the frequency of oral analgesic use from postoperative day 1 to 7. **Results**: The NRS score for neck pain was significantly lower for patients in group M than for those in group H. **Conclusions**: ExLAP is a novel, practical, and minimally invasive surgical technique that may alleviate the postoperative axial pain of patients with cervical myelopathy.

## 1. Introduction

Expansive open-door laminoplasty is widely performed to treat cervical myelopathy [1,2,3,4]. Although it results in favorable outcomes for neurological symptoms, it is associated with several complications, including posterior tissue damage and postoperative axial pain and kyphotic deformity [5,6].

Microscopes or surgical loupes are used to perform delicate and precise techniques during conventional spinal surgery. However, an exoscope, which is a newly developed magnification tool, is equipped with a compact, highly sensitive, high-resolution three-dimensional (3D) digital video camera and 4K large-screen monitor [7,8]. As a result, surgeries that were previously conducted with a microscope can be performed with an exoscope instead. The application of minimally invasive endoscopic surgery, which was developed for focal decompression such as one-level or two-level cervical laminectomy [9,10], to cervical laminoplasty procedures for continuous multi-level decompression is challenging. Therefore, simplifying the conversion from conventional open-door laminoplasty procedures to minimally invasive procedures has been difficult; as a result, conventional methods have been predominantly utilized. Minimizing the surgical invasiveness of cervical laminoplasty can help resolve its associated complications, particularly axial pain. Therefore, we propose exoscopic minimally invasive open-door laminoplasty (exLAP), a novel technique, for cervical myelopathy to alleviate postoperative axial pain.

## 2. Materials and Methods

Between March 2023 and November 2023, a total of 28 patients who underwent open-door laminoplasty at C3–C6 at our institution were reviewed. The inclusion criteria were as follows: patients with cervical spondylotic myelopathy or ossification of the posterior longitudinal ligament (OPLL); K-line-positive OPLL; and multi-level lesions without C7/T1 canal stenosis. The exclusion criteria were K-line-negative OPLL and C7/T1 canal stenosis. Of these patients, 17 underwent exLAP (group M) and 11 underwent conventional Hirabayashi open-door laminoplasty (group H). Detailed demographic data are presented in Table 1. The outcomes of the groups were assessed using numerical rating scale (NRS) scores for neck pain on postoperative days 3, 5, 7, and 14 and for the frequency of oral analgesic use from postoperative day 1 to 7. All data are expressed as the mean ± standard deviation. Statistical analyses were performed using Student’s *t*-test to compare the two groups. *p* < 0.05 was considered significant.

### Surgical Technique for exLAP at C3–C6

**Figure 1:**
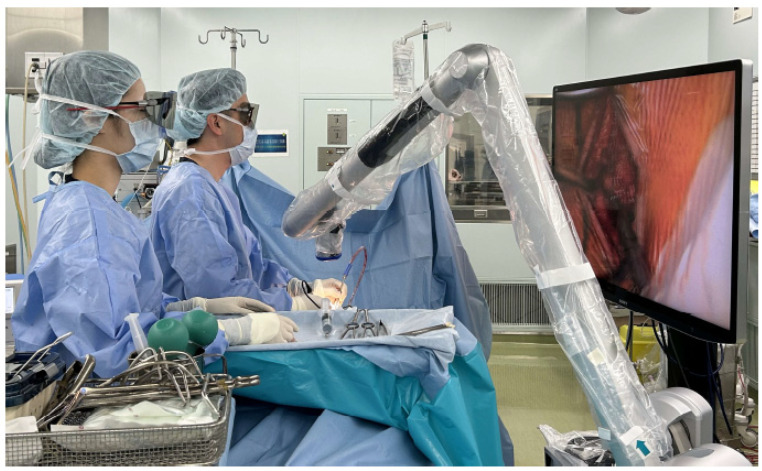
The standard surgical setting during exoscopic minimally invasive open-door laminoplasty using an exoscope. The camera is positioned above the surgical field. The surgeon wears three-dimensional polarized glasses and performs surgery while observing the monitor.

**Figure 2:**
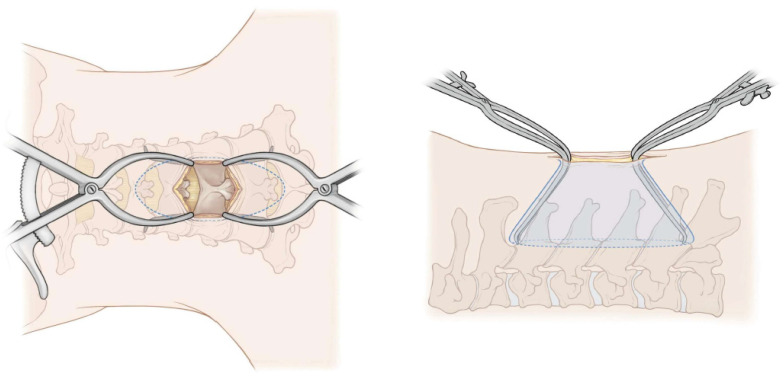
Schematic drawing of the posterior and lateral aspects. A deep Gelpi retractor was used cranially to retract the paraspinal muscles during manipulation of the cranial vertebral laminae and caudally during manipulation of the caudal vertebral laminae.

**Figure 3:**
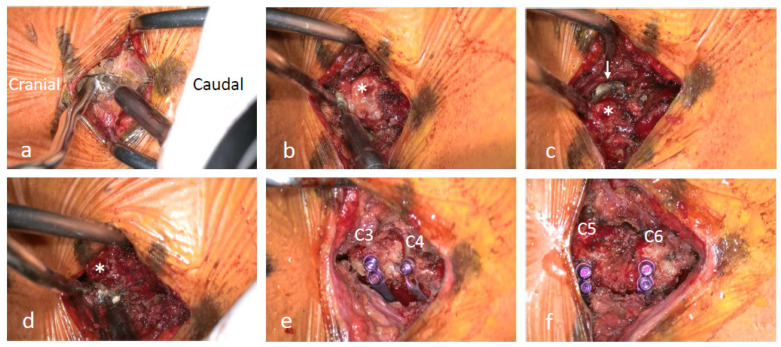
(**a**) The tip of the C3 spinous process is split using a high-speed drill. The attached muscles are not dissected. (**b**) A C2/3 fenestration is performed using a high-speed drill. (**c**) A gutter is created at the hinge side of C3 (↓) using a high-speed drill. (**d**) A gutter is created at the open side of C3 using a high-speed drill. The base of the C3 spinous process (*) is observed. (**e**,**f**) Mini-plates are placed at each lamina.

**Figure 4:**
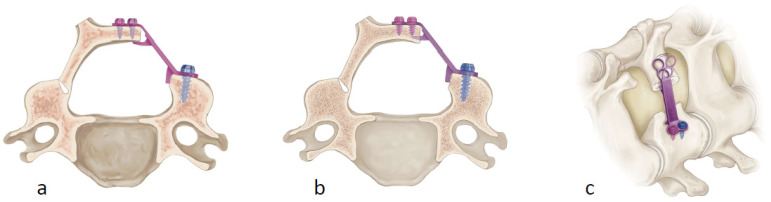
(**a**) The head of the plate is placed to sandwich the lamina. (**b**) The supporting part can be easily inserted into the side of the lamina when the lamina is too thick to be sandwiched. (**c**) The top side of the plate is flexible enough to bend according to the shape of the lamina.

During exLAP, an exoscopic camera was positioned above the surgical field. The surgeon wore 3D polarized glasses and performed the surgery while viewing 3D images on a 4K large-screen monitor (Figure 1). A 30 to 40 mm midline incision was created at the C4/5 level. A deep Gelpi retractor was used to retract the paraspinal muscles to allow for clear visualization (Figure 2). The tips of the spinous processes from C3 to C6 were split using a high-speed drill (Midas Rex; Medtronic, Fort Worth, TX, USA); however, the attached muscles were not dissected (Figure 3a). Bilateral laminae were exposed using an electric knife. The bases of the spinous processes were removed. A fenestration was created between the C2/3 laminae using a high-speed drill, and the ligamentum flavum of C2/3 was dissected using a curved curette (Figure 3b); however, the semispinalis muscles of C2 were not dissected. Using the same procedure, the ligamentum flavum at the C6/7 level was dissected using a curved curette. The orientation of the camera head was frequently adjusted as needed to ensure clear visibility of the manipulated lamina. During manipulation of the C3 and C6 laminae, it was necessary to move the camera head in the cranial and caudal directions. Gutters on both the open and hinge sides from C3 to C6 were created using a high-speed drill in the same manner as that used for the conventional method (Figure 3c,d). Each lamina door was lifted, and mini-plates (OPERA System; Symphony Medical Co., Ltd., Kyoto, Japan) were placed at all of the opened laminae. After screw holes were created in the lamina and lateral mass, the mini-plates were fixed with titanium screws (Figure 3e,f). The mini-plates are designed to be compact for ease of handling, even with a small incision, and there is a mechanism in the head of the plate. Typically, the head of the plate was placed to sandwich the lamina (Figure 4a). When the lamina was thick, the supporting part could be easily inserted into the side of the cancellous bone of the lamina (Figure 4b). The top side of the plate could be easily bent to fit the lamina (Figure 4c). After placing the drainage tube, the incision was irrigated, and each layer of tissue was closed; thereafter, radiography was performed. The patients began ambulation and rehabilitation after the removal of the suction drain on postoperative day 3. They wore a cervical collar for approximately 1 month postoperatively.

## 3. Results

The mean NRS scores for neck pain on postoperative days 3, 5, 7, and 14 were 4.0 ± 2.1, 3.5 ± 1.2, 2.4 ± 1.1, and 1.0 ± 1.1, respectively, for patients in group M; those for patients in group H were 6.6 ± 2.3, 5.1 ± 2.3, 4.9 ± 2.0, and 3.1 ± 1.2, respectively (Table 2). The mean NRS scores for neck pain after surgery were significantly lower for patients in group M than for those in group H at all time points. The mean frequency of oral analgesic use from postoperative day 1 to 7 was 5 ± 3 for patients in group M; however, it was 8 ± 6 for patients in group H (Table 3).

### Representative Case

**Figure 5:**
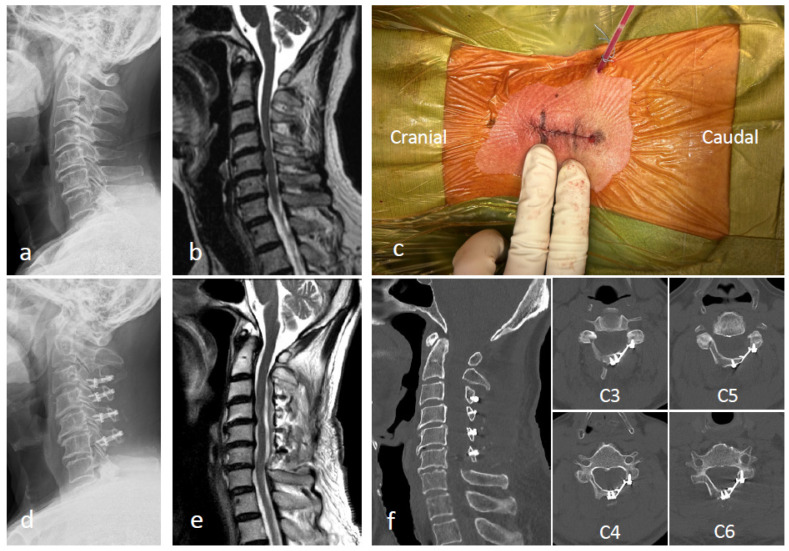
(**a**,**b**) Preoperative radiography and magnetic resonance imaging (MRI) images. (**c**) Exoscopic minimally invasive open-door laminoplasty (exLAP) is performed through a small incision with a length of two fingers. (**d**–**f**) Postoperative radiography, MRI, and computed tomography images after exLAP at C3–C6.

A 73-year-old man was referred to our orthopedic department with numbness, weak handgrip, and gait disturbance. Radiography revealed degenerative findings (Figure 5a). Magnetic resonance imaging (MRI) revealed spinal cord stenosis at C3/4, C4/5, C5/6, and C6/7 (Figure 5b). ExLAP at C3–C6 was performed through a 35 mm skin incision (Figure 5c). Postoperative radiography and MRI revealed a significant reduction in canal stenosis (Figure 5d,e). Postoperative computed tomography revealed that the mini-plates were properly placed (Figure 5f).

## 4. Discussion

**Figure 6:**
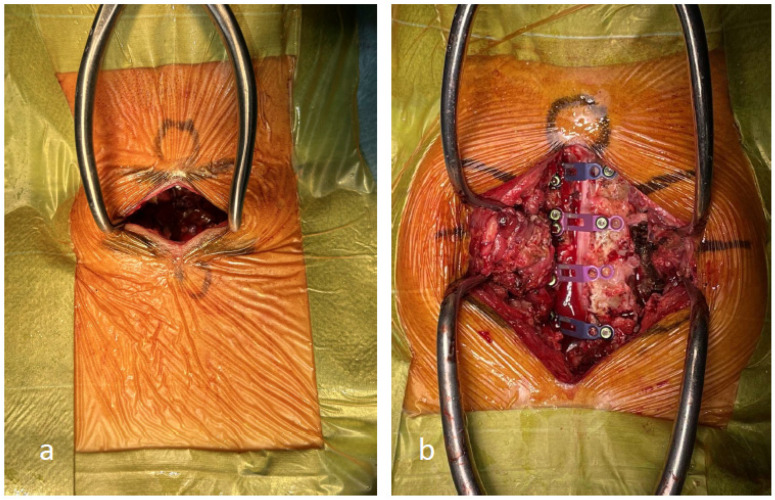
(**a**) The exoscopic minimally invasive open-door laminoplasty incision is less invasive to the posterior tissues than that used for conventional open-door laminoplasty. (**b**) The incision is created during conventional open-door laminoplasty.

Various modifications of open-door laminoplasty can be made to allow for less invasive procedures [11,12,13]. However, complications such as axial pain and kyphotic deformities caused by posterior tissue invasiveness have not yet been resolved. Skip laminectomy is a less invasive procedure for cervical myelopathy that is associated with limited damage to posterior structures [14]. Systematic reviews have shown that skip laminectomy is superior to laminoplasty in terms of axial pain, muscle injury, and complication rates [15,16]. However, skip laminoplasty has disadvantages, such as insufficient decompression and adjacent segmental disorder near the residual lamina. Laminoplasty is advantageous because it allows for continuous and extensive decompression. However, exLAP, which is a novel technique, may resolve these complications because it allows for continuous multilevel decompression of the spinal cord and is less invasive to the posterior tissues than conventional open-door laminoplasty (Figure 6a,b). The goal of exLAP is to achieve the same clinical results with less injury to the surrounding tissues because fewer tissue injuries are correlated with less postoperative axial pain and better outcomes.

At our institution, we perform surgeries using ORBEYE (Sony Olympus Medical Solutions, Tokyo, Japan), which is an exoscope that was developed to address issues associated with the use of conventional microscopes [7,8]. During exoscopic spinal surgery, the camera head is positioned above the surgical field. The long focal length of ORBEYE provides a wide working space between the camera and surgical field and enables dissection through small incisions. Additionally, the flexible camera head allows for a nearly horizontal visual axis that is not achievable with microscopes. Surgeons wear 3D polarized glasses and perform surgery while viewing a 4K large-screen monitor. The use of the large-screen monitor facilitates the sharing of surgical information with the surgical team, including assistant surgeons, instrument nurses, anesthesiologists, and medical students.

**Figure 7:**
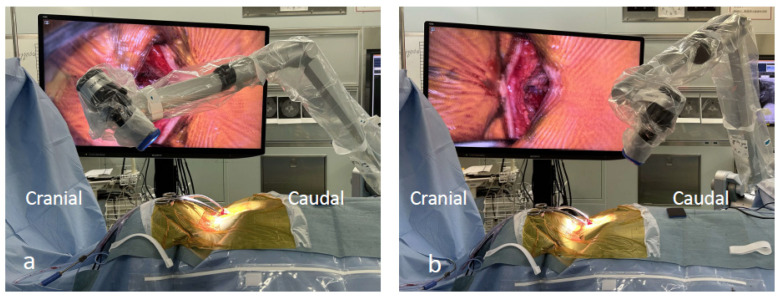
(**a**,**b**) The camera head is adjusted in the caudal direction during manipulation of the C6 lamina; however, it is adjusted in the cranial direction during manipulation of the C3 lamina.

**Figure 8:**
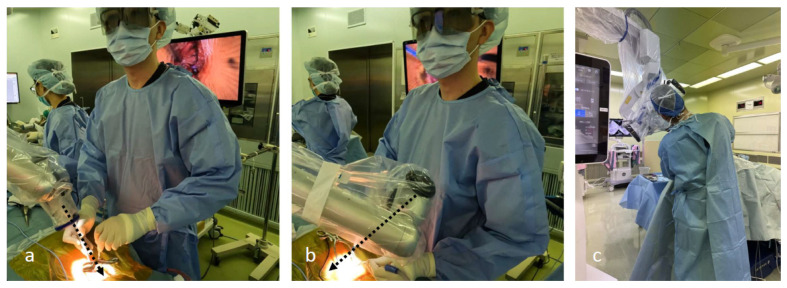
(**a**,**b**) Surgeons can perform surgery while maintaining a comfortable position, regardless of the camera head angle. (**c**) When using a microscope, surgeons must adjust their posture according to the tilt of the microscope.

By finely adjusting the compact camera head of the ORBEYE in the cranial, caudal, left, and right directions, a clear field of view through small incisions can be maintained (Figure 7a,b). The camera head must be tilted significantly during the manipulation of C3 and C6 laminae. However, surgeons can perform this procedure in a comfortable and less fatiguing position than that assumed during microscopic surgery (Figure 8a,b). When using a microscope, surgeons must adjust their posture to align with the base of the microscope, which is uncomfortable (Figure 8c).

Compared with conventional methods, the minimally invasive exLAP approach allows for complete preservation of the intervertebral joint capsule and semispinalis muscle of C2; furthermore, it results in significantly reduced postoperative axial pain during the acute postoperative period. Moreover, the use of oral analgesics tended to be reduced during the acute phase. ExLAP is applicable to all cases previously treated using conventional methods. Although this study was limited to surgical cases at the C3–C6 level, exLAP can be applied to any lamina from C3–C7. However, in cases involving laminoplasty of all five laminae from C3–C7, it may be necessary to widen the skin incision to more than 40 mm. The main advantage of exLAP is the potential to reduce postoperative axial pain, thus providing significant benefits for patients and surgeons who experience axial pain after surgery. After a surgeon gains sufficient experience performing exLAP, a surgical assistant is no longer required. Therefore, eventually, the surgeon can safely perform exLAP alone, similar to endoscopic surgery. Although exLAP is less invasive than conventional methods, it involves manipulation through small incisions guided by a monitor; therefore, there is a learning curve. As hand–eye coordination is required, exLAP is more similar to endoscopic surgery than it is to microscopic surgery. Moreover, because there is a tendency for inadequate orientation, careful attention is required when determining the vertebral body levels and creating gutters. Other disadvantages include the use of an exoscope; however, exLAP can be performed using a microscope. Few individuals experience 3D motion sickness with 3D polarized glasses. Although conventional methods often involve fixation of the open laminae with sutures, the small skin incision used for exLAP makes suture use challenging, thereby necessitating the use of plates and incurring implant costs. Nevertheless, the exLAP approach is less invasive than the conventional Hirabayashi method and can alleviate axial pain.

### Limitations

This study had some limitations. The number of patients was relatively low, and evaluations were performed only during the acute phase. Further research of mid-term and long-term outcomes is necessary.

## 5. Conclusions

ExLAP is a practical and minimally invasive surgical technique for patients with cervical myelopathy that can alleviate postoperative axial pain. Therefore, we believe that exLAP is valuable in clinical practice.

## Figures and Tables

**Table 1:** Demographic data of groups M and H.

Characteristics	Group M (exLAP)	Group H (Conventional)	*p*-Value
Patients (no.)	17	11	
Age (years)	70 ± 11	71 ± 10	0.932
Sex			
Male	13	9	
Female	4	2	
Diagnosis			
CSM	15	7	
OPLL	2	4	
Preoperative lordosis angle (°)	12 ± 10	4 ± 13	0.116
Skin incision length (mm)	34 ± 2	84 ± 14	<0.001
Operative time (min)	89 ± 17	81 ± 31	0.360
Intraoperative blood loss (mL)	37 ± 54	62 ± 54	0.246

Values are presented as means ± standard deviations unless otherwise indicated. Group M underwent exoscopic minimally invasive open-door laminoplasty (exLAP). Group H underwent conventional Hirabayashi open-door laminoplasty. CSM, cervical spondylotic myelopathy; OPLL, ossification of the posterior longitudinal ligament. The lordosis angle was the angle formed by the two tangential lines to the posterior wall of the C2 and C6 vertebrae measured before surgery.

**Table 2:** NRS scores for neck pain from postoperative day 3 to 14.

NRS Scores for Neck Pain	Group M (exLAP)	Group H (Conventional)	*p*-Value
Day 3	4.0 ± 2.1	6.6 ± 2.3	0.004
Day 5	3.5 ± 1.2	5.1 ± 2.3	0.025
Day 7	2.4 ± 1.1	4.9 ± 2.0	<0.001
Day 14	1.0 ± 1.1	3.1 ± 1.2	<0.001

Values are presented as means ± standard deviations unless otherwise indicated. Group M underwent exoscopic minimally invasive open-door laminoplasty (exLAP). Group H underwent conventional Hirabayashi open-door laminoplasty. NRS, numerical rating scale.

**Table 3:** Frequency of oral analgesic use from postoperative day 1 to 7.

	Group M (exLAP)	Group H (Conventional)	*p*-Value
Frequency of analgesic use	5 ± 3	8 ± 6	0.062

Values are presented as means ± standard deviations unless otherwise indicated. Group M underwent exoscopic minimally invasive open-door laminoplasty (exLAP). Group H underwent conventional Hirabayashi open-door laminoplasty.

## Data Availability

The data used to support the findings of this study are available from the corresponding author upon request.
